# Safety and efficacy of peripheral metaraminol infusion in patients with neurological conditions: a single-center retrospective observational study

**DOI:** 10.3389/fneur.2024.1398827

**Published:** 2024-06-03

**Authors:** Pan Han, Yu Zhou

**Affiliations:** General ICU, Second Affiliated Hospital, School of Medicine, Zhejiang University, Hangzhou, China

**Keywords:** metaraminol, peripheral infusion, neurologic patients, hemodynamic stability, safety and efficacy

## Abstract

**Introduction:**

Metaraminol is a sympathomimetic amine vasopressor that can be administrated through a peripheral venous access. However, limited evidence restricts its application in critically ill patients. This study aimed to investigate the safety and efficacy of peripheral metaraminol infusion in patients with neurological conditions.

**Methods:**

Patients who received peripheral metaraminol infusion between May 2019 and April 2022 were recruited. Data on baseline characteristics, clinical parameters, and infusion-related complications were retrospectively collected and analyzed.

**Results:**

273 patients who received metaraminol were enrolled. Of these, 35 (12.8%) patients required central venous catheter insertion due to inability in achieving hemodynamic stability following peripheral metaraminol monotherapy. In 29,574.2 hours of vasopressor infusion, metaraminol infusion resulted achievement of the target blood pressure 73.4% of the time. Meanwhile, adverse events occurred in 5 patients and resolved after local tissue treatment.

**Discussion:**

Metaraminol could provide hemodynamic support and avoid complications associated with a central venous catheter and delay in vasopressor administration. Through careful and close monitoring, peripheral metaraminol infusion is safe and feasible for patients with neurological conditions. Future large-scale, prospective, multicenter studies are needed to evaluate the safety and efficacy of metaraminol infusion through a peripheral intravenous catheter.

## Highlights

- In patients with neurological conditions, peripheral metaraminol infusion is safe and feasible when carefully monitored.
- Metaraminol is a potentially effective vasopressor in terms of pharmacological efficacy and timeliness in mild shock.
- Peripheral metaraminol infusion could avoid complications associated with a central venous catheter and delay in vasopressor administration.

## Introduction

Hemodynamic manipulation and vasopressor infusion are vital and common components of neuro-critical management, especially for the maintenance of cerebrospinal perfusion and neuroprotection. Vasopressors are also administered to avoid secondary injury due to various causes of hypoperfusion. Vasopressor administration conventionally requires the insertion of a central venous access to prevent complications during peripheral infusion. However, insertion and maintenance of a central venous catheter (CVC) or peripherally inserted central catheter (PICC) may result in various complications, including procedural issues, infections, and thrombosis (1). More than 15% of complication rate notably interfere with patient clinical outcome (2).

It is known that metaraminol, a mixed beta-and alpha-adrenergic agonist, indirectly affects norepinephrine secretion and is reportedly effective in managing hypotension during spinal anesthesia and acute myocardial infarction (3, 4). The reason for metaraminol’s effects may be attributed to its similar effects with norepinephrine on hemodynamic variables (4). According to international guidelines, norepinephrine remains the first-line vasopressor for the treatment of most shock subtypes (5, 6). However, studies assessing the efficacy of metaraminol in the critical care setting are limited (5). Compared to CVC and PICC, insertion of a peripheral intravenous catheter (PIC) is faster and easier for medication delivery. Another advantage of metaraminol over other vasopressors is its safety when administered peripherally (7). However, metaraminol infusions in peripheral pathways are currently not well-studied due to the lack of real-world data (7, 8).

The objective of this retrospective study was to investigate the safety and efficacy of peripheral metaraminol infusion in patients with neurological conditions.

## Methods

### Study design and patient selection

This retrospective observational review was conducted in the department of the General Intensive Care Unit (ICU) at a tertiary academic medical center. Patients with cerebrospinal diseases who received metaraminol were recruited between May 2019 and April 2022. Metaraminol was peripherally administered through a PIC to correct hypotension or to increase hemodynamic parameters, depending on the physician’s decision. The exclusion criteria were as follows: age < 18 years, pregnancy, with contraindications to metaraminol, bolus injection of metaraminol, vasopressor infusion through a CVC or PICC, metaraminol combined with other vasopressors, and incomplete data. This study was approved by the review board of the Second Affiliated Hospital of Zhejiang University Medical School.

### Data collection

Metaraminol was peripherally administered with a standard concentration of 1.0 mg/mL via an ≥18G PIC, which was placed in the bilateral external jugular and upper extremity veins. The infusion rate was titrated to reach the target blood pressure (BP) and was restricted to not exceed 20 mg/h. A CVC was inserted if hemodynamic instability persisted based on the physician’s judgment. Baseline characteristics, primary diagnosis, indications, vascular risk factors, and clinical results were recorded. Vascular risk factors included vascular diseases, hypertension, diabetes, smoking, and hyperlipidemia. Infusion parameters of metaraminol and other vasopressors were investigated, including maximum rate, duration, and frequency of administration. Infusion complications were defined as site pain, erythema, phlebitis, tissue necrosis, and ischemia. Nurses observed for any complications during routine nursing care. Once adverse events occurred, the PIC was transferred to an alternative location. Injured local tissue was primarily managed with external application of medications and surgical intervention, if necessary.

### Statistical analysis

Data were obtained from electronic medical records and analyzed using SPSS statistics for windows version 24.0 (IBM Corp, Armonk, N.Y., USA) and GraphPad Prism version 9.0 for windows (Graph Pad software, San Diego, California, USA). Categorical variables were expressed as proportions. Continuous variables were described as median and interquartile ranges.

## Results

**Figure 1:**
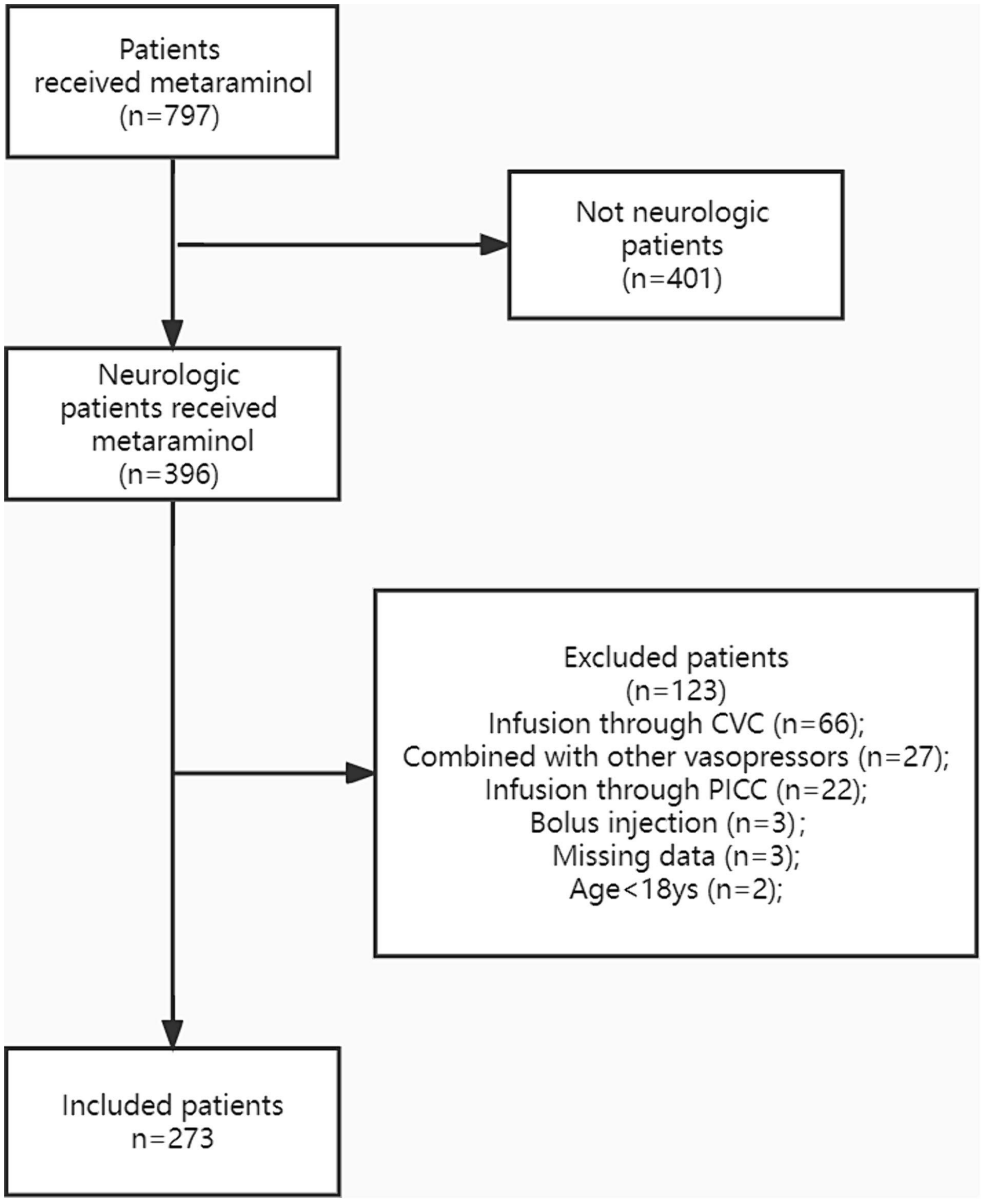
The study population was presented in the flow diagram. CVC, central venous catheter; PICC, peripherally inserted central catheter.

Overall, 273 patients were enrolled in the study (Figure 1). The baseline characteristics of the cohort are summarized in Table 1. The mean age of patients was 64 years (IQR: 55–71), and 188 (68.9%) were men. The median BMI was 22.9 (IQR: 20.4–25.3), and 221 (81.0%) patients had at least one comorbidity. Meanwhile, 200 (73.3%) patients had multiple risk factors, including hypertension (105, 38.5%), coronary and cerebrovascular diseases (63, 23.1%), and diabetes (40, 14.7%). The average APACHE II and Glasgow coma scores were 21.0 (IQR: 16–27) and 8 T (IQR: 4 T–10 T), respectively. The most common primary diagnosis was traumatic brain injury (*n* = 65, 23.8%) followed by cerebral hemorrhage (*n* = 53, 19.4%) and spinal cord injury (*n* = 36, 13.2%).

**Table 1:** Baseline characteristics of the patients.

Baseline characteristics	Patients (*n* = 273)
Age (years), median (IQR)	64 (55–71)
Male, *n* (%)	188 (68.9%)
BMI, median (IQR)	22.9 (20.4–25.3)
APACHE II score, median (IQR)	21.0 (16.0–27.0)
GCS, median (IQR)	8 T (4 T-10 T)
Diagnosis, *n* (%)	
Traumatic brain injury	65 (23.8)
Cerebral hemorrhage	53 (19.4)
Spinal cord injury	36 (13.2)
Subarachnoid hemorrhage	34 (12.5)
Ischemic stroke	34 (12.5)
Neoplasm	17 (6.2)
Intracranial infections	10 (3.7)
Others	24 (8.8)
Duration of hospitalization (days), median (IQR)	20.7 (9.6–23.9)
Duration of ICU stay (days), median (IQR)	13.6 (4.4–17.2)
Comorbidity, *n* (%)	
None	52 (19.0)
≥1 comorbidity	221 (81.0)
Vascular risk factor, *n* (%)	
None	73 (26.7)
≥1 risk factor	200 (73.3)
Clinical result, *n* (%)	
Transfer	126 (46.2)
Discharge against medical advice	73 (26.7)
Discharge complied with medical advice	62 (22.7)
Death	12 (4.4)

IQR, interquartile range; BMI: Body mass index; APACHE II, acute physiology and chronic health evaluation II; GCS, Glasgow Coma Score; ICU, intensive care unit.

Metaraminol was commonly administered through an 20G PIC. The most common location of PIC insertion was at the right antecubital area, which was also the first choice for initial insertion. Additionally, 35 (12.8%) patients required CVC insertion because peripheral metaraminol monotherapy failed to achieve the target BP (Table 2). Furthermore, CVC was inserted in 19 (7.0%) patients due to inadequate or failed peripheral access. Central venous pressure was routinely monitored in the 54 aforementioned patients. At the same time, the CVC was removed in 55 (20.1%) patients to avoid complications; peripheral metaraminol monotherapy was initiated in these patients. Of the 55 patients, central venous access in 41 (15.0%) was achieved outside of our ICU.

**Table 2:** Metaraminol infusion.

Variables	Patients (*n* = 273)
Indication for metaraminol, *n* (%)	
To increase cerebral perfusion	96 (35.2)
To prevent hypotension	82 (30.0)
Both	95 (34.8)
Maximum rate of infusion (ml/h), median (IQR)	8.7 (5.0–10.0)
Duration of metaraminol infusion (hours), median (IQR)	79.3 (18.9–106.6)
Infusion <24 h, *n* (%)	83 (30.4)
Infusion 24-48 h, *n* (%)	58 (21.2)
Infusion 48-96 h, *n* (%)	59 (21.6)
Infusion 96-168 h, *n* (%)	38 (13.9)
Infusion >168 h, *n* (%)	35 (12.8)
Duration of vasopressors infusion (hours), median (IQR)	108.3 (28.0–134.3)
Infusion <24 h, *n* (%)	60 (22.0)
Infusion 24-48 h, *n* (%)	55 (20.1)
Infusion 48-96 h, *n* (%)	64 (23.4)
Infusion 96-168 h, *n* (%)	41 (15.0)
Infusion >168 h, *n* (%)	53 (19.4)
Metaraminol repeated infusion times, *n* (%)	
Once	251 (91.9)
≥twice	22 (8.1)
Patients with metaraminol monotherapy to achieve target BP, *n* (%)	238 (87.2)
Patient-hours with metaraminol monotherapy to achieve target BP, *n* (%)	21697.8 (73.4)
CVC placed prior to stopping vasopressors infusion, *n* (%)	
Inability to achieve target BP	35 (12.8)
Failed or inadequate peripheral access	19 (7.0)
CVC removed prior to stopping vasopressors infusion, *n* (%)	55 (20.1)
Complication, *n* (%)	
Yes	5 (1.8)
None	268 (98.2)

IQR, interquartile range; BP, blood pressure; CVC, central venous catheter.

**Figure 2:**
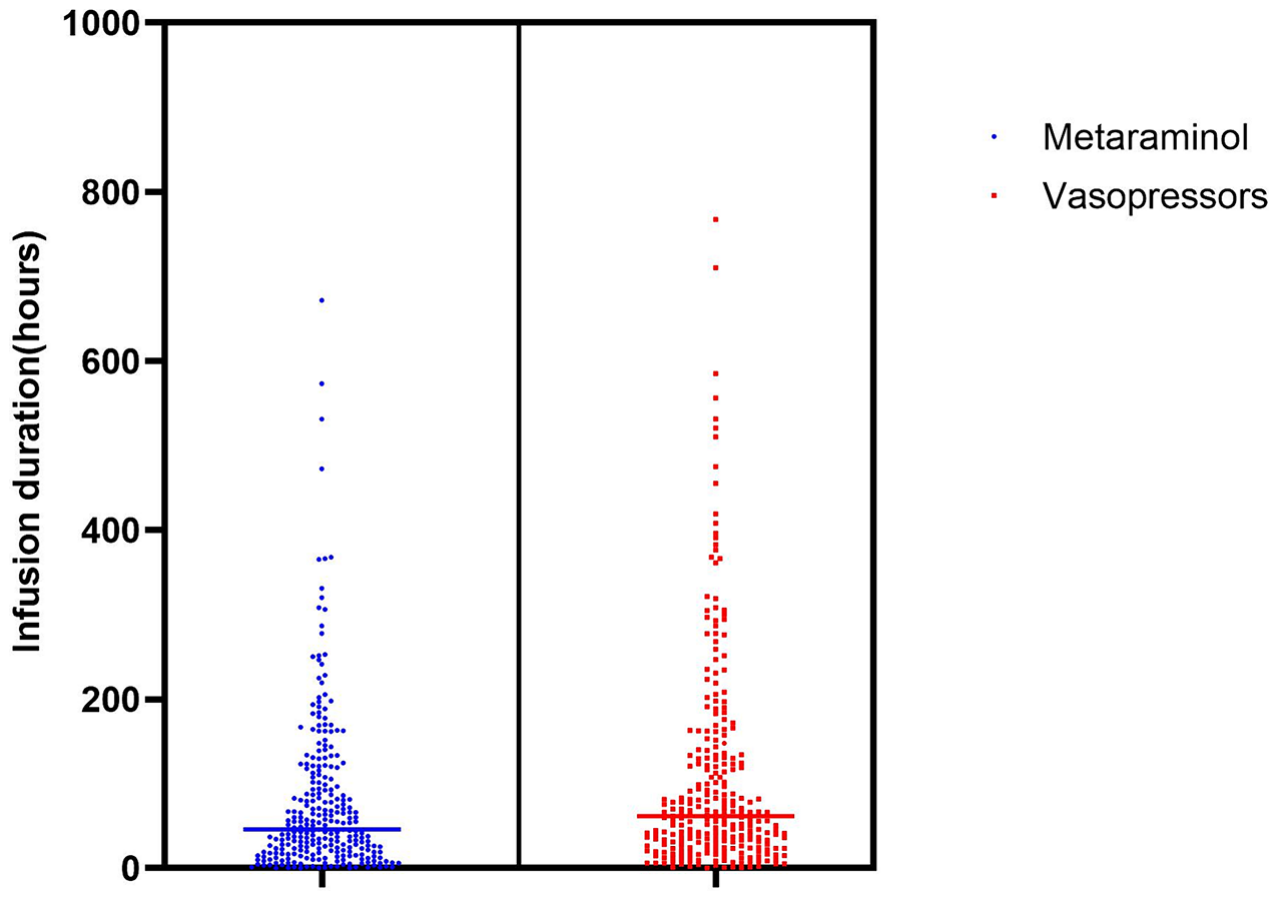
Duration of vasopressors infusion.

For accurate BP measurements, all patients had an arterial line placed before and during metaraminol infusion. To increase cerebral perfusion (*n* = 96, 35.2%) was the most common indication for metaraminol infusion. Meanwhile, 82 (30.0%) patients received metaraminol to prevent hypotension (for all causes). The average peak rate of metaraminol infusion was 8.7 mg/h (IQR: 5.0–10.0 mg/h), and the median duration was 79.3 h (IQR: 18.9–106.6 h) (Figure 2). The target BP was achieved in 83 (30.4%) patients within 24 h, and the total duration of infusion was sustained up to 48 h in 141 (51.6%) patients. In the remaining 132 (48.4%) patients, administration of metaraminol persisted beyond 48 h, and the longest duration was 671.9 h. Furthermore, 22 (8.1%) patients received repeated peripheral metaraminol administration of up to 5 times. During 29,574.2 patient-hours of vasopressor infusion, the target BP was achieved 73.4% of the time with peripheral metaraminol monotherapy. Metaraminol administration maintained the target BP in 55 (20.1%) patients after the CVC was removed.

Adverse events included 5 (1.8%) cases of phlebitis and 1 (0.4%) case of intravenous infiltration, both of which were resolved by local tissue treatment, including topical administration of mucopolysaccharide polysulfate cream (Table 3). One patient developed concurrent phlebitis and infiltration at the left forearm and antecubital fossa. Meanwhile, change in the dose, or type of vasopressor or CVC removal was not necessary in the 5 patients who developed phlebitis. With the support of peripheral metaraminol monotherapy, the average maximum infusion rate was 7.4 mL/h (IQR: 5.5–9.0 mL/h), and the average duration was 88.4 h (IQR: 54.5–131.6 h). All patients who developed adverse events restarted metaraminol infusion via another peripheral access; the external jugular vein was deemed as the safest location. There were no serious adverse events, and no patient required surgery.

**Table 3:** Infusion complications.

Variables	Patients (*n* = 5)
Age (years), median (IQR)	74.4 (69.0–80.5)
Male, *n* (%)	3 (60)
BMI, median (IQR)	21.2 (16.5–27.3)
APACHE II score, median (IQR)	22.6 (16.0–28.5)
GCS, median (IQR)	6.4 T (4.5 T-9.0 T)
Duration of hospitalization (days), median (IQR)	11.4 (6.0–16.5)
Duration of ICU stay (days), median (IQR)	9.0 (5.2–13.4)
Comorbidity, *n* (%)	
None	0
≥1 comorbidity	5
Vascular risk factor, *n* (%)	
None	0
≥1 risk factor	5
Tissue injury, *n* (%)	
Phlebitis	5
Infiltration	1
Location, *n* (%)	
Upper arm	4
Forearm	1
Antecubital fossa	1
Maximum rate of metaraminol infusion, median (IQR)	7.4 (5.5–9.0)
Duration of metaraminol infusion (hours), median (IQR)	88.4 (54.5–131.6)
Duration of vasopressors infusion (hours), median (IQR)	88.4 (54.5–131.6)
Clinical result, *n* (%)	
Discharge complied with medical advice	3
Discharge against medical advice	1
Transfer	1
Death	0

IQR, interquartile range; BMI: Body mass index; APACHE II, acute physiology and chronic health evaluation II; GCS, Glasgow Coma Score; ICU, intensive care unit.

## Discussion

To our knowledge, this is the largest study to investigate the safety and efficacy of peripheral metaraminol infusion. CVC insertion, which is a common procedure in the ICU, is performed depending on the severity of a patient’s disease as well as the requirement for monitoring and treatment (9). Among the various indications for CVC placement, vasopressor support is the most common to prevent the risk of adverse events during infusion (10). However, CVC insertion may increase the risk of related complications, especially under emergent conditions. Moreover, to reduce the incidence of CVC-associated bloodstream infections, shortening the indwelling time is recommended (11); hence, vasopressor infusion through a PIC has been recommended (12, 13). It is extremely rare for patients with a PIC to develop thrombosis, and PIC-related bloodstream infections occur 40 times less often than CVC-related bloodstream infections (14). Ricard et al. conducted a prospective observational study comparing vasopressor infusion in critically ill patients through PIC and CVC (15). Although the complication rate was higher with PIC than with CVC, the main difference was the incidence of minor complications, including difficulty of insertion, and erythema. In an earlier study, patients with PICs had a higher incidence of phlebitis, while patients with CVCs had a higher incidence of major complications (16). Moreover, Lewis et al. reported that in 202 patients who received peripherally administered vasopressors, the extravasation rate was 4%, and all events were conservatively managed without further interventions (12). Notably, vasopressor therapy is not recommended as an absolute indication for CVC insertion. Our cohort revealed similar result. In our study, the indication for CVC insertion in 35 patients was infusion of other vasopressors. The adverse event rate was 2.2%, paralleling the low complication rate of other peripherally administrated vasopressors in previous studies. In all cases, the infusion site was transferred, and all complications were resolved by conservative management. However, due to the non-occurrence of severe complications in our study, it is unreasonable to make the conclusion that vasopressor infusion through a PIC is safer than that through a CVC. Regardless, peripheral delivery permitted faster initiation of therapy and a shorter time to achieve hemodynamic stability in patients.

Early commencement of vasopressors contributes to early reversal of hypotension and restoration of organ perfusion. Observational studies revealed that delayed initiation of vasopressors was associated with increased mortality in sepsis (17, 18). The 2021 Surviving Sepsis Campaign guideline emphasizes the early initiation of vasopressors to restore BP (6). Due to CVC placement requiring expertise and time, initiating vasopressors via a PIC is recommended until a CVC can be secured. Natalini et al. compared metaraminol with norepinephrine in patients with septic shock and demonstrated that there was no difference in the resulting hemodynamic parameters or acid–base status (4). Despite having a similar hemodynamic effect with norepinephrine, metaraminol is not the first-line vasopressor in the ICU. In contrast, Sardaneh et al. conducted a retrospective observational study and advocated that metaraminol should be a first-line peripheral vasopressor in the ICU (19). Patients who received metaraminol monotherapy had a shorter vasopressor infusion time and a shorter length of stay in the ICU and hospital. Metaraminol monotherapy could also be effective in patients with less severe shock. In our study, peripheral metaraminol infusion provided hemodynamic augmentation and reversed hypotension in 80.6% of patients, consequently avoiding CVC placement, and its related complications. Norepinephrine was the vasopressor commonly administered following CVC insertion. However, once the CVC was removed, metaraminol could also achieve adequate hemodynamic support. In 73.4% of the total vasopressor infusion time, peripheral metaraminol monotherapy achieved the target BP, suggesting that peripheral metaraminol administration was effective and should be selected when CVC insertion was delayed. The efficacy presented no significant difference between lower BMI (<22.9) and higher BMI (>22.9). In higher BMI (average 29.0) patients with hypotension during caesarean section, metaraminol is at least non-inferior to phenylephrine reported by McDonnell et al. (3). Additionally, peripheral metaraminol infusion could be an alternative vasopressor in patients suspected of having catheter-related bloodstream infections once the CVC was removed. Overall, metaraminol is a potentially effective vasopressor in terms of pharmacological efficacy and timeliness.

Compared with peripheral norepinephrine, it is believed that peripheral metaraminol is associated with a lower risk of complications from infusion (20). However, limited evidence does not suggest that peripheral metaraminol infusion is safer that norepinephrine infusion. Delaney et al. compared initial vasopressor infusions via PIC and CVC (10). In their cohort, skin necrosis, or ischemia did not occur in any pathway, suggesting that vasopressor infused through a PIC had a low risk of tissue injury and was not associated with an increased risk of death. Many complex factors associated with extravasation can be divided into patient-related, infusion-related, and institution-related factors (12). Infusion-related factors include the duration of infusion, concentration of infusion, infusion rate, and location, and size of the PIC. A systematic review of 318 adverse events caused by peripheral vasopressor infusion showed that local tissue injury and extravasation were associated with a distal PIC location and long-term infusion (>2 h) (21). Peripheral administration of vasopressors supposedly increases local vasoconstriction and tissue hypoperfusion. Short duration and proximal locations were supposed to be the crux of the matter. In our study, most patients (260, 95.2%) received metaraminol for >2 h. The average and maximum infusion duration were 79.3 h and 671.9 h, respectively. Although the infusion duration in our cohort mostly exceeded 2 h, we noted a very low complication rate. In patients who developed complications, the average, and maximum infusion duration were 88.4 h and 164.2 h, respectively, which were longer than those in previous reported studies. Meanwhile, the average, and maximum infusion rates were 7.4 mg/h and 10 mg/h, respectively, which were slower than those of the whole cohort. It was noteworthy that all 5 patients who developed complications had more than one preexisting comorbidity and risk factor, which may have influenced the occurrence of the complications. Due to the low overall complication rate, identifying baseline characteristics associated with metaraminol infusion-related complications were difficult. Combined with those from aforementioned studies, our results confirm that a PIC is a safe and feasible infusion pathway. However, limited data made it difficult to reveal the association between the peripheral infusion parameters and adverse events.

## Limitations

There are still several shortcomings in our study. First, this is a single-center observational study; clinical application of metaraminol should be promoted with judicious care. Second, the study’s retrospective design may lead to the inaccuracy of data acquisition. Patient selection, missing information, documentation errors, and other bias were inherent in this study. Third, due to the limited sample sizes and low complicate rate, it was difficult to measure the clinical parameters and reveal relationships. Therefore, it is necessary to conduct large-scale and multicenter prospective studies and randomized clinical trials in the future.

## Conclusion

Our study demonstrates that through close monitoring and observation, peripheral metaraminol infusion is safe and feasible in neurological patients. Metaraminol infusion through a PIC could achieve adequate hemodynamic support and prevent complications associated with CVC insertion and delay in vasopressor administration. Prospective, randomized, and multi-center trials are required to evaluate the safety and efficacy of metaraminol infusion through a PIC.

## Data availability statement

The raw data supporting the conclusions of this article will be made available by the authors, without undue reservation.

## Ethics statement

The studies involving humans were approved by the Second Affiliated Hospital of Zhejiang University Medical School. The studies were conducted in accordance with the local legislation and institutional requirements. The ethics committee/institutional review board waived the requirement of written informed consent for participation from the participants or the participants' legal guardians/next of kin because due to the retrospective nature of the study.

## Author contributions

PH: Writing – original draft, Writing – review & editing. YZ: Data curation, Writing – review & editing.
